# PDX1 is the cornerstone of pancreatic β-cell functions and identity

**DOI:** 10.3389/fmolb.2022.1091757

**Published:** 2022-12-15

**Authors:** Nour Ebrahim, Ksenia Shakirova, Erdem Dashinimaev

**Affiliations:** ^1^ Center for Precision Genome Editing and Genetic Technologies for Biomedicine, Pirogov Russian National Research Medical University, Moscow, Russia; ^2^ Moscow Institute of Physics and Technology (State University), Dolgoprudny, Russia

**Keywords:** PDX1, pancreas, β-cells, insulin, diabetes

## Abstract

Diabetes has been a worldwide healthcare problem for many years. Current methods of treating diabetes are still largely directed at symptoms, aiming to control the manifestations of the pathology. This creates an overall need to find alternative measures that can impact on the causes of the disease, reverse diabetes, or make it more manageable. Understanding the role of key players in the pathogenesis of diabetes and the related β-cell functions is of great importance in combating diabetes. PDX1 is a master regulator in pancreas organogenesis, the maturation and identity preservation of β-cells, and of their role in normal insulin function. Mutations in the PDX1 gene are correlated with many pancreatic dysfunctions, including pancreatic agenesis (homozygous mutation) and MODY4 (heterozygous mutation), while in other types of diabetes, PDX1 expression is reduced. Therefore, alternative approaches to treat diabetes largely depend on knowledge of PDX1 regulation, its interaction with other transcription factors, and its role in obtaining β-cells through differentiation and transdifferentiation protocols. In this article, we review the basic functions of PDX1 and its regulation by genetic and epigenetic factors. Lastly, we summarize different variations of the differentiation protocols used to obtain β-cells from alternative cell sources, using PDX1 alone or in combination with various transcription factors and modified culture conditions. This review shows the unique position of PDX1 as a potential target in the genetic and cellular treatment of diabetes.

## 1 Introduction

Diabetes mellitus (diabetes) is a chronic metabolic disease characterized by high blood glucose levels and associated with impaired insulin secretion, insulin action, or both (Weir et al., 1990; Kerner et al., 2014). According to the World Health Organization, more than 400 million people are living with diabetes worldwide, prompting global efforts to stop the rise of the disease. Many approaches are being used in the treatment of diabetes, ranging from conventional methods, like the use of pharmaceutical compounds, to more advanced approaches such as gene and cellular therapies (Ryan et al., 2005; Wong et al., 2010; Sorli and Heile, 2014; Hering et al., 2016; Loretelli et al., 2020). However, many of them fall short as substitutes for the sophistication of human β-cells. Therefore, in pursuit of understanding the pathogenesis of diabetes, we must understand the key players in the molecular mechanisms of β-cells. One of these key players is pancreatic and duodenal homeobox 1 (PDX1), also known as insulin-promoting factor 1 (IPF1).

Although *PDX1* is predominantly expressed in β-cells and some δ-cells of the islets of Langerhans, it is also expressed in the gastrointestinal tract (duodenum, stomach, pancreas), and the central nervous system during development (Perez-Villamil et al., 1999; Fagerberg et al., 2014). *PDX1* is one of the early-expressed genes during pancreas development and one that persists through β-cell maturation. Studies have revealed its role in the normal development of the pancreas by regulating the fate and propagation of pancreatic precursor cells (Jonsson et al., 1994). Furthermore, *PDX1*-expressing progenitors give rise to both exocrine and endocrine lineages, hence *PDX1* loss-of-function mutations lead to arrested development of the exocrine compartment and an underdeveloped endocrine compartment, since endocrine lineage is also affected by *NGN3*-expressing progenitors (Oliver-Krasinski et al., 2009). Mutations in the *PDX1* gene cause pancreas agenesis, maturity-onset diabetes of the young 4 (MODY4) and other pancreatic dysfunctions (Jonsson et al., 1994; Stoffers et al., 1997). PDX1 is also linked to diabetes pathogenesis; in type 1 diabetes (T1D) PDX1 autoantibodies have been detected, while in type 2 diabetes (T2D), *PDX1* expression levels are compromised (Li et al., 2010; Guo et al., 2013; Abreu et al., 2021). These data solidify the role of PDX1 as a master regulator of embryonic pancreatic formation, in both exocrine and endocrine compartments, and most importantly in the maturation and development of β-cell function.

## 2* PDX1* gene structure and regulation of expression

PDX1 was first described as a nuclear endodermal protein expressed in the epithelium of the duodenum and the pancreas (Wright et al., 1989). Later, PDX1 was linked to β-cells’ specific expression of insulin by binding and transactivating the insulin gene promoter (Ohlsson et al., 1993). PDX1 also activates other pancreas-associated genes like those for SST (somatostatin), GCK (glucokinase), IAPP (islet amyloid polypeptide), RFX6 (regulatory factor X6), HNF1B (HNF1 homeobox B), and even PDX1 itself (Wang et al., 2018).

The *PDX1* gene is highly conservative among different species. It is composed of two exons spanning a region of 6 kb on chromosome 13 and encoding a protein of 283 amino acids without any known splice forms (Stoffel et al., 1995). One exon encodes for the NH2- terminal region containing a DNA activation domain and the other exon encodes for the COOH- terminal region and the homeodomain region that contains three helixes and harbors a nuclear localization signal responsible for DNA binding (Inoue et al., 1996; Melloul et al., 2002; Schwitzgebel et al., 2003; Stanojevic et al., 2004).

**FIGURE 1:**
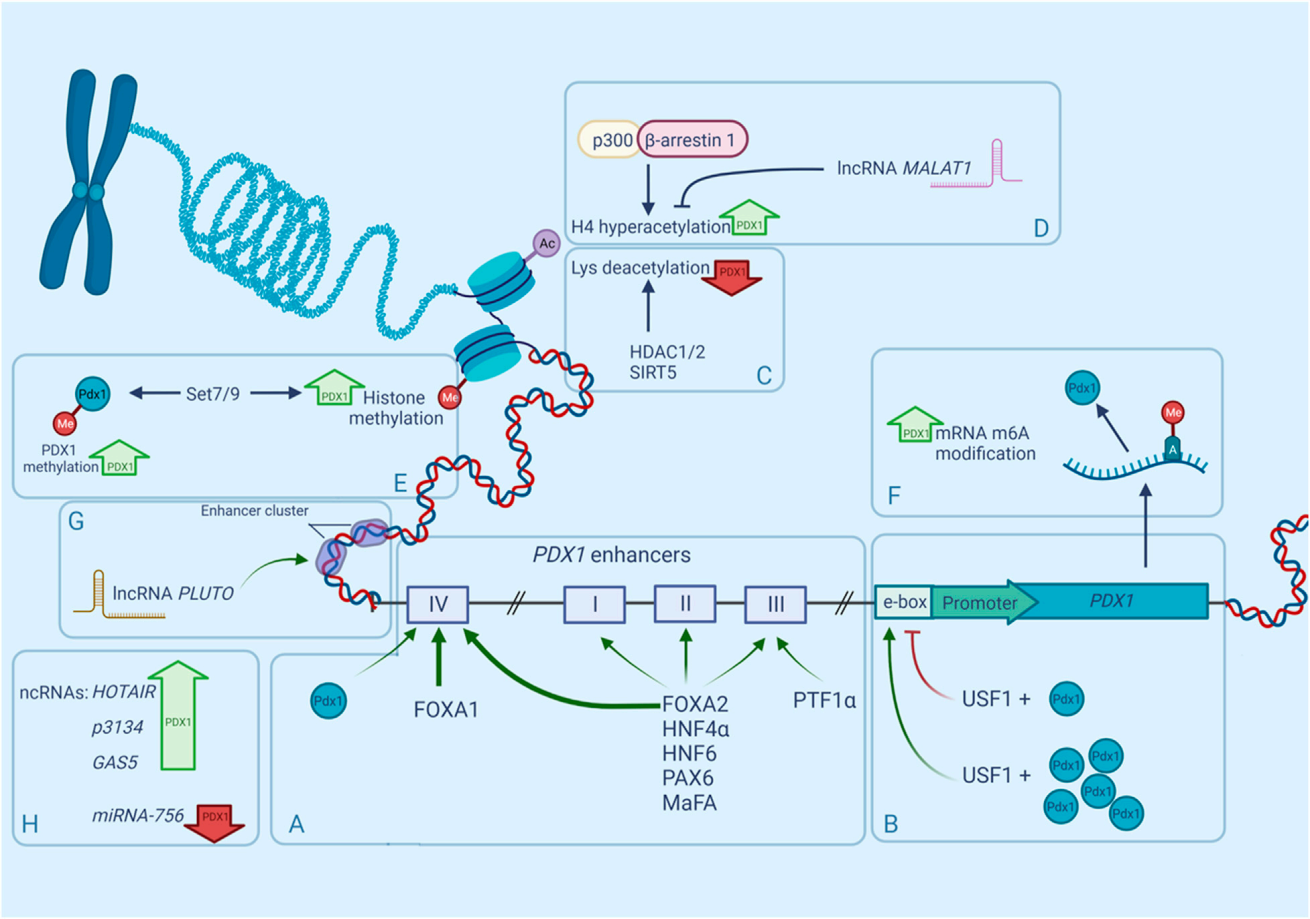
Regulation of mammalian PDX1 gene by multiple factors. Expression of *PDX1* is controlled by numerous epigenetic factors such as nucleosome positioning, histone methylation, histone acetylation, accessibility of enhancers and non-coding RNAs. PDX1 expression can be upregulated (bold green upward arrows) or downregulated (bold red downward arrows) by any of these factors. **(A)**. Distant enhancers known as areas I-IV, participate in PDX1 transcription regulation due to their ability to bind transcription factors and boost gene expression. Same TFs can bind to multiple areas with different affinities, for example FOXA1/2 occupy area IV more efficiently (bold green arrows) than other areas (narrow green arrows). **(B)**. regulation of *PDX1* through upstream regulatory factors (USF) and E-box. **(C)**. interaction of *PDX1* and histone deacetylases. **(D)**. interaction between β-arrestin-1, P300 and their role in PDX1 regulation. **(E)**. PDX1 recruitment to Lys methyltransferase Set7/9. **(F)**. RNA modification in *PDX1* regulation. **(G)**. **(H)**. role of ncRNAs in PDX1 regulation.

Although *PDX1* has only one promoter (Figure 1), it is regulated by a large number of distant enhancers mainly located in the 5′-flanking region of the gene (Sharma et al., 1996; Wu et al., 1997; Campbell and Macfarlane, 2002). Those enhancers were first discovered and characterized by studying the nuclease hypersensitive sites (HSS). HSS are markers of transcription factor (TF) binding sites, and three of them have been identified in the approximate area between −3,000 and +180 bp of the mouse *Pdx1* gene. HSS1 (−2,560 to −1880), has demonstrated an ability to control the β-cell-specific expression of *PDX1* (Wu et al., 1997). Further investigation of the HSS1 site showed that it can be divided into three subdomains: area I (−2,694 to −2,561 bp), area II (−2,139 to −1958 bp), and area III (−1879 to −1799 bp). The most distant *PDX1* enhancer is located near −6,000 bp in the mouse gene and near −8,300 bp in the human gene and is known as area IV (Gerrish et al., 2004). Areas I, III, and IV are very conserved between mice, humans, and chickens, sharing 78–89% similarity, whereas area II is present only in mammals (Gerrish et al., 2000; Gerrish et al., 2004). Analyses of the enhancer region have shown that areas I–III contain binding sites for important transcription factors participating in pancreatic organogeneses, such as HNF1α, FOXA2, HNF6, PAX6, and MaFA (Dassaye et al., 2016), moreover, areas I–II contain binding sites both for transcriptional activators and inhibitors of *PDX1* (Gerrish et al., 2004). Further analyses of enhancer functions show that areas I, II, and IV are capable of maintaining the expression of the β-cell-specific reporter in transfection assays independently of each other (Marshak et al., 2000; Gerrish et al., 2004), and together areas I and II can induce strong *PDX1* expression in β-cells (Van Velkinburgh et al., 2005). Although area III seems to be less important and does not drive β-cell-selective activity, it contributes to *PDX1* expression during embryogenesis through its binding of the *PTF1*α transactivator (Gerrish et al., 2000; Wiebe et al., 2007). Furthermore, the removal of areas I–III *in vivo* leads to decreased expression of *PDX1* and impairs the formation of the pancreas in the early development stages in mice (Fujitani et al., 2006)—an outcome similar to pancreatic agenesis in homozygous loss-of-function PDX1 phenotypes.

As was mentioned before, Area II is unique to mammals and has multiple essential functions both in early endocrine cell specification and postnatal β-cell maturation. Deletion of this locus does not affect pancreas development or size, but it does change islet cell composition. Homozygous absence of Area II in *PDX+/-*pancreas (PDX1ΔII/−) results in a large decrease in endocrine progenitors, abnormal β-cell specification, and subsequent hyperglycemia in newborn mice (Yang et al., 2017). Furthermore, the *PDX1* deficient condition associated with the PDX1ΔII/− state caused a significant alteration in the proportion of α- and β-cells, shifting the balance towards the α-cell type, probably because of the inhibited PDX1-mediated repression of the *ARX* gene expression—a main α-cell fate inductor (Collombat et al., 2007; Yang et al., 2017). These data demonstrate that area II of the *PDX1* enhancer participates in maintaining a proper α- and β-cell balance in the islets. Of note, PDX1 repression of the *ARX* gene is mediated by co-expression of the Groucho-related gene 3 (*GRG3*), a member of the Groucho family of co-repressors (Metzger et al., 2014), but interactions of the *GRG3*, *ARX*, and *PDX1* area II enhancers are yet to be investigated.

Like area II, area IV is not essential for normal organogenesis of the pancreas, but it regulates postnatal *PDX1* expression and β-cell functions and growth. Area IV has binding sites for both FOXA1 and FOXA2 which are pioneer TFs of several foregut-derived organs, including the pancreas (Figures 1A). These TFs occupy area IV more efficiently than other areas and their bindings are heavily dependent on developmental time, and increases with age (Gao et al., 2008). Interestingly, mice with area IV mutations exhibited sexually dimorphic phenotypes: affected diabetic males with reduced PDX1 levels manifested hyperglycemia at weaning time *versus* phenotypically normal females (Spaeth et al., 2017). Spaeth et al. (2017) also suggested that PDX1 may autoregulate area IV during weaning which could explain why PDX1 levels increase in this period of development (Stolovich-Rain et al., 2015). Additionally, area IV of the *PDX1* enhancer is tissue-specific and is upregulated by HNF3β and NEUROD/b2 strictly in islet cells (Naya et al., 1997; Kaestner, 2000). This region is also influenced by glucocorticoids, which reduce *PDX1* expression by interfering with HNF3β activity (Sharma et al., 1997).

Besides its occupation of the *PDX1* enhancer, transactivation of the gene by FOXA2 is also augmented by FAM3A—a mitochondrial protein that enhances ATP production. Yang et al. (2020) studied the role of mitochondria in regulating *PDX1* expression in pancreatic β-cells and demonstrated that FAM3A-induced ATP production elevates cellular Ca^2+^ levels, which results in the release of activated calmodulin to function as a co-activator of FOXA2, thus stimulating *PDX1* gene transcription. This is one of the possible mechanisms that link mitochondrial dysfunction with insulin deficiency under diabetic conditions.

After transcription, PDX1 cooperates with FOXA1 and FOXA2 (FOXA1/2) to promote downstream gene regulation. Generally, FOXA1/2 bind to the enhancers of target genes, assisting increased deposition of histone H3 lysine four mono-methylation (H3K4me1) that leads to chromatin relaxation and accessibility for other TFs, a process known as enhancer priming (Lee et al., 2019). A recent study has shown that FOXA1/2 recruitment to primed enhancers before pancreatic lineage induction is independent of PDX1, whereas, in contrast, FOXA1/2 binding with unprimed enhancers requires cooperation with PDX1 (Geusz et al., 2021). This indicates that full chromatin accessibility and enhancer activation during β-cell development are heavily reliant on PDX1.

Another group of enhancers are the E-boxes (enhancer boxes)—short DNA sequences that share a signature motif CANNTG (N for any nucleotide) and act like protein-binding sites (Massari and Murre, 2000). During pancreas genesis, PDX1 activates its promoter through the proximal (−104/−99 bp) E-box motif in the *PDX1* promoter region, creating a positive autoregulatory loop (Melloul et al., 2002). Upstream stimulatory factor 1 (USF1) occupies that E-box motif and has been proven to be crucial for the autoregulation of *PDX1* (Amemiya-Kudo et al., 2011). First, it was discovered that USF1 forms a complex with PDX1, and together they activate the expression of the *PDX1* gene, however further investigation revealed that USF1 has a dose-dependent repressing effect on the PDX1 promoter, but is successively abrogated in a higher concentration of PDX1 (Spohrer et al., 2017) (Figure 1B).

Interestingly, both PDX1 and USF1 have been identified as substrates for protein kinase CK2 (Meng et al., 2010; Lupp et al., 2014). Spohrer et al. (2017) have shown that CK2 is a negative regulator of USF1-dependent *PDX1* transcription because of the CK2 phosphorylation of USF1, which strengthened the USF1 interaction with the PDX1 protein.

All these combined findings indicate the importance of the conserved enhancer elements for *PDX1* expression both in pancreas development and in the maintenance of β-cells.

## 3 Epigenetic control of PDX1 function

Epigenetic modifications are an important part of the gene expression machinery that operate transcription by changing the state of the chromatin. PDX1 regulates pancreas development and β-cell maintenance with the help of various recruited coregulators. Among them are numerous epigenetic modifiers that participate in DNA methylation, histone modification, chromatin remodeling, and ncRNA operation (Spaeth et al., 2016).

Genome sequencing of the islets of T2D donors has revealed that the *PDX1* gene is heavily methylated in diabetes (Volkov et al., 2017). Interestingly, high levels of glucose promote methylation of *PDX1* in isolated T2D islets, decreasing the expression of the gene even further (Yang et al., 2012). Different levels of glucose also affect the chromatin landscape shifting of PDX1 in β-cells. According to Mosley and Ozcan (2004) in the presence of low glucose levels, PDX1 is located in the nuclear periphery, where it interacts with the histone deacetylases HDAC1 and HDAC2, allowing them to target the insulin promoter, producing condensed chromatin and consequently reducing insulin gene expression (Figure 1C). When the concentration of glucose becomes high, PDX1 translocates to the nucleoplasm where it interacts with histone acetyltransferase p300. P300 causes the hyperacetylation of histone H4 in the insulin promoter, thus stimulating insulin gene expression (Mosley et al., 2004) (Figure 1D). However, in contrast to the studies of Mosley and others (Rafiq et al., 1998; Elrick and Docherty, 2001), later experiments did not find any evidence of glucose-dependent changes in the localization of PDX1 (Spohrer et al., 2017).

Recently it has been shown that β-arrestin-1 participates in the engagement of p300 to PDX1 (Figure 1D). β-Arrestin-1 and β-arrestin-2 are intracellular signaling proteins that participate in the sensitization of many G protein-coupled receptors (GPCRs) (Pierce and Lefkowitz, 2001) or act independently (Shukla et al., 2011). Tissue-specific knockout of β-arrestin-2 in mouse β-cells causes metabolic deficits, including impaired insulin secretion and reduced glucose tolerance (Zhu L. et al., 2017). Surprisingly, β-cell-specific β-arrestin-1 knockout in mice fed a standard chow did not show similar deficits (Barella et al., 2019), but severe impairment in glucose tolerance in mice on an obesogenic diet was reported (Barella et al., 2021). Further investigation showed that β-arrestin-1 deficiency in β-cells leads to reduced *PDX1* expression because of the lack of β-arrestin-1 complexes with p300, which normally promote *PDX1* transcription (Barella et al., 2021).

The nucleosome state heavily affects the availability of chromatin for TFs. The SWI/SNF family members forming part of the ATP-dependent chromatin remodeling complex are key regulators of nucleosome positioning (Euskirchen et al., 2012). Like HDACs, the SWI/SNF complex interacts with PDX1 in a glucose-dependent manner (McKenna et al., 2015). The mammalian SWI/SNF complex contains ATPase subunits, either BRG1 or BRM. In low glucose, PDX1 interacts with the BRM:SWI/SNF complex, allowing it to repress its target genes *INS1*, *SLC2A2*, and *UCN3*. Whereas in high glucose, PDX1 binds to the BRG1:SWI/SNF complex, which, in contrast, enhances the expression of these genes (Kim and Kulkarni, 2020).

Class III HDACs also known as sirtuins (SIRTs) are involved in the regulation of pancreas development and glucose homeostasis. SIRT1 promotes β-cell formation by boosting the transcription of *PDX1* through the deacetylation of FOXA2 on the promoter of the *PDX1* gene (Wang et al., 2013). Oppositely, SIRT5 downregulates the transcription of *PDX1* through H4K16 deacetylation of its promoter region (Ma and Fei, 2018).

PDX1 co-modifiers are also involved in histone methylation and demethylation. For example, PDX1 recruits Lys methyltransferase Set7/9 to the INS gene, where it performs H3-K4 methylation and thus activates transcription of the gene in mouse β-cells (Deering et al., 2009). Set7/9 also takes part in *PDX1* expression regulation (Figure 1E). The β-cells specific knockout of Set7/9 results in the downregulation of *PDX1* and other important β-cell genes like *MAFA*, *GCK*, and *GLUT2*, causing a shift from insulin production to increased proliferation (Maganti et al., 2015; Jetton et al., 2021). Furthermore, Set7/9 seems to be important for the PDX1 protein itself: it has been shown that Set7/9 methylates the N-terminal residue Lys-131 of PDX1, augmenting *PDX1* transcriptional activity, which is important for the maintenance of normal β-cell function and glucose homeostasis (Maganti et al., 2015).

RNA modification is a relatively newly discovered mechanism of gene expression regulation that also takes part in *PDX1* regulation. One of the most abundant RNA modifications is the methylation of the adenosine in N6-position-m6A (Figure 1F) (Frye et al., 2018). It was shown that the m6A landscape of T2D islets significantly differs from the landscape of healthy islets—the mRNA pool in T2D islets is hypomethylated. Artificial depletion of m6A levels in EndoC-βH1 (immortalized human β-cell line) cells results in G0-G1 cell cycle arrest and impaired insulin secretion due to downregulation of the insulin/IGF1–AKT–PDX1 pathway, decreasing the AKT phosphorylation and PDX1 protein levels (De Jesus et al., 2019).

Non-coding RNAs (ncRNA) are other key players in gene regulation. More than 1,000 cell-type-specific long non-coding RNAs (lncRNA) have been identified both in human and murine pancreatic islets (Moran et al., 2012; Benner et al., 2014), most of which are located outside of genes, but near the islet-specific chromatin domains and protein-coding regions (Moran et al., 2012).

Frequently, enhancer clusters are targets of lncRNAs. An enhancer cluster, otherwise defined as a superenhancer, is a group of enhancers in close genomic proximity that are bound by multiple TFs (Pott and Lieb, 2015). LncRNAs affect the binding of TFs with pancreatic-enhancer-cluster-associated genes, changing their affinity for targets (Pasquali et al., 2014; Akerman et al., 2017). Knockdown of islet-specific lncRNAs identified their ability to modulate gene expression and, consequently, insulin secretion in human β-cells. It was shown that in T2D islets several lncRNAs were significantly altered compared to healthy islets (Akerman et al., 2017; Sathishkumar et al., 2018).

*PDX1* is regulated by many lncRNAs, but only a few are well-characterized. One of them is *HI-LNC71*, also known as *PLUTO* (*PDX1* locus upstream transcript). *PLUTO* regulates *PDX1* transcription by affecting the 3D contacts between the enhancer cluster and the *PDX1* promoter (Figures 1G). Although lncRNA sequences generally are not conserved across different species, *PLUTO* regulation of *PDX1* has been confirmed for both mouse and human orthologs, underlining the possible importance of that lncRNA in the modulation of *PDX1* expression (Akerman et al., 2017).

Circulating *lncRNA-p3134* also participates in the regulation of *PDX1* and other β-cell-associated TFs (Figure 1H). It has been shown that overexpression of *lncRNA-p3134* in the mouse pancreatic β-cell line MIN6 upregulates *Pdx1*, *MAFA*, and Glut2 expression levels and increases glucose-stimulated insulin secretion consistent with the upregulation of insulin-associated TFs. Moreover, in the condition of high glucose exposure this overexpression partially reversed the inhibitory effect of glucotoxicity on *PDX1* expression, and, as a consequence, the glucose-stimulated insulin secretion (GSIS) function was restored (Ruan et al., 2018). *PDX1* and *MAFA* expression are also affected by *GAS5* (growth arrest-specific transcript 5) (Jin et al., 2017; Esguerra et al., 2020) and *HOTAIR* lncRNAs (Zhu, 2020). *GAS5* is a key regulatory factor in mammalian cell growth, proliferation, and apoptosis (Lander et al., 2001). It is known for repressing the glucocorticoid receptor function (Kino et al., 2010) and its low levels in human serum are associated with T2D (Carter et al., 2015). Knockdown of lncRNA *GAS5* expression resulted in decreased expression of *PDX1* and *MAFA* (Jin et al., 2017), and a similar effect was observed for *HOTAIR* knockdown (Zhu, 2020). Under glucocorticoid-caused *GAS5* downregulation, both PDX1 and NKX6-1 levels were affected (Esguerra et al., 2020).

Although the ncRNA *MALAT1* is associated with the regulation of alternative splicing (Tripathi et al., 2010) (Figure 1D), recently it has been reported that *MALAT1* decreases the expression of *PDX1* by suppressing histone acetylation of the *Pdx1* promoter in MIN6 cells (Ding et al., 2020).

RNA-dependent regulation of *PDX1* expression also involves short non-coding RNAs. For example, *miRNA-765* targets the *PDX1* gene and reduces its products on both the mRNA and protein levels, which results in impaired survival and function of pancreatic β-cells (Zheng et al., 2021).

Taken together, this data indicates that the participation of ncRNAs in *PDX1* regulation makes them a potential target for T2D treatment procedures and use in protocols directing transdifferentiation into β-cells.

## 4 Role of PDX1 in pancreas organogenesis and β-cells maturity

The human pancreas is composed of exocrine and endocrine compartments. The orchestration of differentiation and acquisition of cell identity requires the intricate and coordinated expression of different transcription factors during pancreas development.

### 4.1 Role of PDX1 during early pancreatic development

**FIGURE 2:**
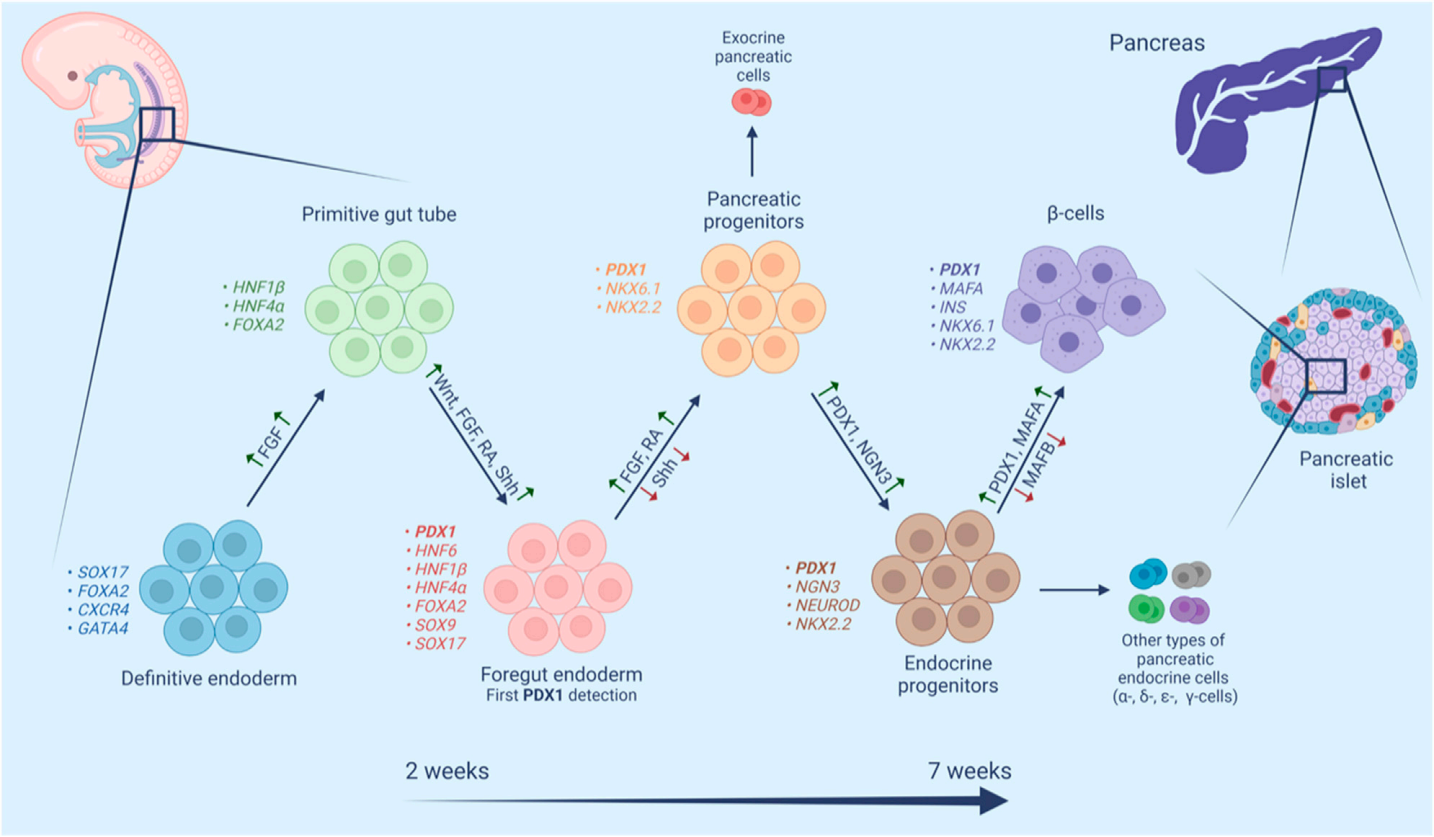
Schematic representation of human β-cell maturation stages. β-Cell differentiation is a complex multistage process. In each stage cell populations have distinctive markers and require both activation (green upward arrows) and repression (red downward arrows) of different signaling pathways, transcription factors, hormones and small molecules.

The pancreas originates from a flat sheet of cells known as the definitive endoderm (Figure 2). The definitive endoderm shares a common progenitor with mesoderm called mesendoderm. Specification of the endoderm is affected by many factors including Wnt and Nodal signaling, both of which favor endoderm formation at high levels (Lickert et al., 2002; Vincent et al., 2003). The definitive endoderm then folds into a primitive gut tube, which in turn develops into foregut endoderm (Wells and Melton, 1999). Fibroblast growth factor (FGF), retinoic acid (RA), and the Wnt, and Sonic hedgehog signaling (Shh) pathways have been implicated in foregut endoderm formation (Spence and Wells, 2007). At this stage, the expression of PDX1 is detected making it one of the earliest transcription factors to be expressed in the developing pancreas (Jennings et al., 2013). The fate of the foregut endoderm is determined by Shh signaling. Repression of Shh is necessary for the development of the foregut endoderm into pancreatic progenitor, meanwhile, expression of Shh causes loss of pancreatic gene expression (Apelqvist et al., 1997; Kim et al., 1997). Repression of Shh marks the primary pancreas specification at around 29 days post-conception in humans. The molecular events underlying the primary specification are still not clear, one study has suggested that secretion of FGF2 by the notochord represses Shh signaling and thereby induces expression of pancreatic genes including *PDX1* (Hebrok et al., 1998). Moreover, RA signaling at this stage induces several transcription factors including PDX1 (Kumar et al., 2003). The early expression of *PDX1* is accompanied by the expression of *SOX9*, *GATA4*, *FOXA2*, and *SOX17*, however, approximately 30–33 days post-conception, the expression of *SOX17* is later lost and replaced with that of *NKX6.1*. This expression profile of *PDX1*, *SOX9*, *NKX6.1*, and *FOXA2* is a hallmark of multipotent pancreatic progenitors (Jennings et al., 2013). Pancreatic progenitors can give rise to a variety of pancreatic cells including exocrine and endocrine cells (Aydin et al., 2020). NKX6.1 is a critical transcription factor in pancreatic β-cell function and proliferation. NKX6.1 expression increases throughout pancreas development where it engages in endocrine commitment and later become restricted to β-cell (Al-Khawaga et al., 2018). The balance between NKX6.1 and PTF1A determines the fate of pancreatic progenitors into exocrine and endocrine commitment. Studies have suggested an antagonistic mechanism between these 2 TFs, in which overexpression of NKX6.1 reduced PTF1A expression and subsequently acinar cell generation, while reduced expression of NKX6.1 showed high expression of PTF1A and a substantial reduction in endocrine progenitors (Schaffer et al., 2010). The simultaneous co-expression of PDX1 and NKX6.1 in pancreatic progenitors warrants their commitment to mono-hormonal, glucose-responsive β-cells (Aigha and Abdelalim, 2020; Memon and Abdelalim, 2020). On the other hand, pancreatic progenitors expressing PDX1 without NKX6.1 (PDX1^+^/NKX6.1^-^) develop into poly-hormonal β-cell that fail to function properly *in vivo* (Aigha and Abdelalim, 2020). Interestingly, Memon et al. (2021) have demonstrated that PDX1^-^/NKX6.1^+^ progenitors can give rise to insulin producing glucose-responsive β-cells.

By the end of the embryonic period, NGN3 detection marks the endocrine commitment and the appearance of pancreatic endocrine cells including fetal β-cells with subsequent detection of nuclear NKX2.2, NKX6.1, PDX1, FOXA2, and ISL1 (Jennings et al., 2013). NGN3 is downregulated by the Notch signaling pathway and upregulated by a cross-regulatory transcription factor network composed of four transcription factors (SOX9, HNF6, HNF1b, FOXa2) (Apelqvist et al., 1999; Lynn et al., 2007). Studies have shown that PDX1 is a member of this cross-regulatory network and it participates directly in the expression of *NGN3*, which leads to the upregulation of *NEUROD1* pushing the cells toward endocrine differentiation (Oliver-Krasinski et al., 2009). A different study has shown a cooperative connection between PDX1 and HNF6 in the early pancreatic bud and their role in the activation of *NGN3*, subsequent endocrine specification, and the functional maturation of β-cells (Henley et al., 2016). On the other hand, activation of *NGN3* leads to the activation of *HES-1* in neighboring cells, which inhibits endocrine differentiation through its actions on *NGN3* (Jensen et al., 2000). This lateral inhibition model ensures that some progenitors undergo a programmed downregulation of *NGN3* and *PDX1*, which is required for the formation of the exocrine compartment of the pancreas (Kaneto et al., 2007).

PDX1 also plays a crucial role in cell fate determination, this is manifested in several aspects. The relationship between PDX1 and MAFA is one example, MAFA upregulates *PDX1* expression in adult islets (Samaras et al., 2003), however, it is not necessary for primary *PDX1* induction. Meanwhile, PDX1 is expressed in cells before MAFA and it induces MAFA^−^MAFB^+^ to MAFA^+^MAFB^−^ transition in the late stages of embryogenesis (Nishimura et al., 2006). This transition contributes to the differentiation and maturation of pancreatic β-cells while concurrently pushing cells away from α-cell commitment, where MAFB plays a pivotal role (Nishimura et al., 2006; Hang and Stein, 2011; Conrad et al., 2016).

The role of PDX1 in pancreatic fate determination is also seen by its repression of the intestinal progenitors in the gut tube. PDX1 in cooperation with SOX9 represses the intestinal master transcription factor CDX2 in the pancreatic domain of the gut tube, thus preventing intestinal fate conversion while upregulating pancreas-restricted TFs like PTF1α and NKX6.1 (Shih et al., 2013; Shih et al., 2015). Moreover, PDX1 also downregulates hepatic-specific genes by its actions on *HNF1α*. The human *HNF4α* gene contains two promoters *P1* and *P2* that drive the expression of two sets of isoforms, *HNF4α* one to six and *HNF4α* seven to nine, respectively. The *P1*- and *P2*-derived isoforms have different functions and different affinity to *HNF4α* targets (Thomas et al., 2001). PDX1 upregulates *HNF1α P2* transcripts with weaker transactivation potential than *P1* transcripts, causing them to compete for targets and eventually reducing the activation of target liver-specific genes by the *P1* transcripts (Donelan et al., 2015).

### 4.2 Role of PDX1 in pancreatic islets

During embryogenesis, β-cells are multihormonal immature cells with high proliferative ability; the features of functional β-cells are gained after birth, particularly around weaning time, as demonstrated by their robust GSIS with simultaneous loss of proliferative ability (Sun et al., 2021). The maturation of β-cells is controlled by many transcription factors, the most important of which is MAFA—a TF that plays a role in insulin granule synthesis and secretion (Hang et al., 2014). PDX1 induces MAFB to MAFA transition in later stages of embryogenesis (discussed above). Furthermore, PDX1 plays a significant role in the maturation and identity perseveration of β-cells after birth as it controls the activation of insulin and other genes responsible for glucose sensing and metabolism like GLUT2 and glucokinase (Kaneto et al., 2008). In addition, Kropp et al. (2018) showed that the cooperative function of PDX1 and osteocalcin 1 (OC1) is necessary for the specification and differentiation of pancreatic endocrine cells and postnatal islet maturation.

Interestingly, some degree of β-cell heterogeneity has been reported in normal adult islets (Szabat et al., 2009). Nasteska et al. (2021) have shown that β-cells exist in different stages of maturation in adult islets. Adult islets house highly plastic, immature β-cells with low expression of *PDX1* and *MAFA* alongside highly mature β-cells with high expression of both *PDX1* and *MAFA*. Presumably low levels of PDX1 promote insulin expression during the maturation process, while higher PDX1 levels in mature cells maintain β-cells in differentiated state promoting increased expression of GLUT2 and glucokinase (Szabat et al., 2009). This heterogeneity of β-cells is crucial for proper islet function since it constitutes a reservoir of cells that can be mobilized under stress conditions (Nasteska et al., 2021).

There’s combinatorial effect of PDX1 and FOXA2 in postnatal maturation of β-cells, we mentioned before that both TFs cooperate to promote downstream gene regulation (Lee et al., 2019). *MAFA*, *Ins1*, and *Slc2a2* are among the genes activated by both TFs in the islets and they are involved β-cells maturation, function, insulin secretion and MODY (Bastidas-Ponce et al., 2017). One study generated mice with reduced expression of both TFs which resulted in upregulation of genes responsible for α-cell fate like *MAFB* with alterations in β-cells numbers and developed hyperglycemia at weaning time. Moreover, the deletion of *PDX1* in mature β-cells led to them losing multiple β-cell markers, combined with either loss of hormone expression or to their adapting of a glucagon-secreting ⍺-cell phenotype since PDX1 binds and inhibits several ⍺-cell genes like *MAFB*, as was mentioned before (Ritz-Laser et al., 2003; Gao et al., 2014).

## 5 PDX1 and diabetes

Mutations of the *PDX1* gene lead to different outcomes that affect pancreas structure, functions or both. Homozygous mutations in the *PDX1* gene and other mutations that impair the functionality of the PDX1 protein during embryonic development cause pancreas agenesis in mice and humans and eventually lead to fatal perinatal hyperglycemia (Jonsson et al., 1994; Stoffers et al., 1997; Hui and Perfetti, 2002). While heterozygous mutations in *PDX1* cause maturity-onset diabetes of the young 4 (MODY4) (Abreu et al., 2021). MODY four is a rare monogenic subtype of diabetes mellitus, it is caused by different various mutations in *PDX1* gene and its transactivation domain (Anik et al., 2015; Deng et al., 2019; Abreu et al., 2021; Yoshiji et al., 2022).

Studies have shown that a decrease in *PDX1* expression in *PDX1* knockout mice concurred with poor maturation of β-cells after birth, impaired expression of several β-cells genes, and the appearance of poly-hormonal cells within the islets (Bastidas-Ponce et al., 2017; Spaeth et al., 2017; Jara et al., 2020). In mature β-cells, pdx1 haploinsufficiency leads to diabetes in mice and humans through adapting ⍺-cell phenotype (discussed above).

In fact, a lot of diabetes phenotypes are associated with mutations in the *PDX1* gene. The nature of the phenotype and severity of the condition depends on the type and location of the mutation. Missense mutations in the transactivator domain of *PDX1* can reduce the ability of the PDX1 protein to activate the expression of its target genes during β-cell development and maturation. As an example of the severity of different mutations, the substitution Pro-Thr in the 33 position (P33 T) causes a greater impact on β-cell formation and function than Cys-Arg in the 18 position (Wang et al., 2019). The P33 T mutation not only impairs binding with DNA targets and transcriptional activation functions, but also predisposes to reduced birth weight, miscarriage, and early postnatal death (Gragnoli et al., 2005). Proline insertions have also been shown to be pathogenic and associated with MODY (Karim et al., 2005; Elbein et al., 2006). Several cases of the *PDX1*-mutant MODY are reported, where glutamic acid in position 178 of PDX1 was substituted with other amino acids (Schwitzgebel et al., 2003; Nicolino et al., 2010; Abreu et al., 2021). Glutamic acid in that position in the homeodomain is evolutionarily conserved among several species, and this mutation seems to decrease PDX1 half-life, which could prevent the proper self-activation of PDX1 and consequently decreases protein levels (Schwitzgebel et al., 2003; Abreu et al., 2021). In some cases, different point mutations in the *PDX1* gene impair endocrine progenitor and β-cell development, leading to the downregulation of several PDX1 target genes responsible for insulin synthesis and secretion, which gives rise to non-functional differentiated β-cells with poor responses to glucose changes (Wang et al., 2019).

PDX1 is also linked to both type 1 and type 2 diabetes pathogenesis; in type 1 diabetes (T1D) PDX1 autoantibodies have been detected (Li et al., 2010). Interestingly, these autoantibodies can be used in screening high-risk population susceptible for developing T1D (Donelan et al., 2013). While in type 2 diabetes (T2D), *PDX1* expression levels are compromised (Li et al., 2010; Guo et al., 2013; Abreu et al., 2021). One study found an enrichment of T2D-associated SNPs in *PDX1* occupied sites located in the intronic regions of *TCF7L2* and *HNF1B*. *Hnf1β* is involved in controlling proliferation and survival of multipotent pancreatic progenitors and deletion of the gene causes pancreatic hypoplasia (De Vas et al., 2015), while TCF7L2 has been identified as the locus conveying the highest risk for developing T2DM (McCarthy and Zeggini, 2009). Mutations in their cis-regulatory regions of these two genes predispose to diabetes (Wang et al., 2018). A recent study has found that selected genetic SNPs in *PDX1* and *MC4R* could modify the risk of T2D (Wang et al., 2021).

A number of studies have found a connection between DNA methylation of *PDX1* and reduced activity in T2D islets (Liu et al., 2021). Yang et al. (2012) found out that PDX1 was one of 15 genes with CpG islands within the promoter that were methylation-susceptible in T2D (Yang et al., 2012). DNA methylation leads to reduced levels of PDX1 protein and mRNA, resulting in impaired expression of both GLUT2 and insulin and causing development of hyperglycemia (Ahlgren et al., 1998). Under high glucose concentration, DNA methylation level may increase abnormally which results in decreased insulin secretion and subsequently leads to diabetes (Pinzon-Cortes et al., 2017).

Normal pancreas development with β-cell formation and maturation is a complex multilayered process that can provide a guideline for the induction of insulin-producing cells from non-β cells using various transcription factors in a way mimicking the natural process.

## 6 Role of PDX1 in reprogramming different cell types into pancreatic β-cells

The most promising way to synthesize β-cells from non-β-cells appears to be by mimicking the natural process in which β-cells are developed during pancreas genesis. Based on evidence from years of studies of developmental biology, β-like-cells or at least insulin-producing cells, can be obtained from pluripotent cells, multipotent cells, or mature cell types. Since PDX1 is a master regulator of β-cell differentiation and maturation, a lot of differentiation approaches use PDX1 as the main factor or as a part of multifactorial protocols. In this section, we will review the research in which PDX1 has been used to obtain insulin-producing β-like cells from non-β-cells (Table 1).

**TABLE 1:** Summary of studies using PDX1 in obtaining β-cells (full version Supplementary Material).

Study	Cell source	Transcription factors	Small molecules	Outcome
Differentiated adult cells				
(Ferber et al. 2000)	mouse liver cells	PDX1	N/A	Insulin secretion
(Ber et al. 2003)	mouse liver cells	PDX1	N/A	Induction of pancreatic Exocrine and endocrine genes, insulin secretion
(Imai et al., 2005, Kaneto et al., 2005, Cao et al., 2004, Tang et al., 2006)	mouse liver cells	PDX1-VP16 PDX1	N/A	Expression of pancreatic markers, insulin secretion
(Wang et al. 2007)	mouse liver cells	PDX1-NGN3	N/A	Insulin secretion, near normal GSIT function
(Zhou et al. 2008)	mouse pancreatic exocrine cells	PDX1- MAFA-NGN3	N/A	Adoption of β-cells morphology and marker expression, insulin secretion
(Banga et al. 2012)	mouse hepatic duct-like cells	PDX1-MAFA- NGN3	N/A	Adoption of β-cells morphology and marker expression, insulin secretion
(Cim et al. 2012)	rat liver cells	PDX1-NGN3-MAFA	N/A	Expression of insulin mRNA
(Hickey et al. 2013)	mouse gall bladder epithelial cells	PDX1-MAFA-NGN3	retinoic acid, dibenzazepine	Upregulation of β-cells genes, insulin secretion
(Akinci et al. 2013)	rat pancreatic exocrine cells and hepatocytes mouse hepatocyte-derived small cells	PDX1-MAFA-NGN3	DAPT, BIX-01294, NECA	Increased expression of β-cell markers, insulin secretion
(Chen et al. 2014)	mouse intestine crypts cells	PDX1-MAFA-NGN3	N/A	Adoption of β-cells morphology, insulin secretion
(Cardinale et al. 2015)	human biliary tree stem cells	PDX1	bFGF, PDX-1 peptide	Increased expression of β-cells markers, insulin and C-peptide secretion
(Lima et al. 2016)	human exocrine pancreatic cells	PDX1-NGN3-MAFA-PAX4	ITS, 5-aza-2′-deoxycytidine, sodium butyrate, SB431542, Y27632, betacellulin, exendin-4, nicotinamide	Insulin packaging and secretion
(Xiao et al. 2018)	mouse pancreatic alpha cells	PDX1-MAFA	N/A	Increased β-cell mass, insulin secretion
Embryonic stem cells				
(Xu et al. 2013)	mouse ESCs	PDX1-MAFA- NEUROD-NGN3	β-mercaptoethanol, activin A, retinoic acid, ITS, bFGF, EGF, N2, B27, nicotinamide	Increased expression of β-cells markers, insulin secretion
(Salguero-Aranda et al. 2016)	mouse ESCs	increased *PDX1* expression by small molecules	DETA-NO, valproic acid, P300 inhibitor C646, β-mercaptoethanol	Increased expression of β-cells markers, glucose responsivity, insulin secretion
Induced pluripotent stem cells				
(Saxena et al. 2016)	human iPSCs	PDX1-MAFA- NGN3	Activin A, wnt3A, bFGF, BMP4, VEGF, noggin, FGF10, KGF, EGF, B27, ascorbic acid, KAAD-cyclopamine, retinoic acid, Y-27632, vitamin A, T3, Alk5 inhibitor, dibenzazepine	Adoption of β-cells morphology, insulin secretion
(Rajaei et al. 2018)	human iPSCs	PDX1	activin A, Wnt3a, KGF, EGF, SB431542, B27, KAAD cyclopamine, retinoic acid, noggin, IBMX	Insulin and C-peptide secretion, adoption of β-cells morphology
Mesenchymal stem cells				
(Yuan et al. 2010)	rat bone marrow-derived MSCs	PDX1	N/A	Acquisition of β-cells phenotype, insulin secretion
(He et al. 2011)	human umbilical cord MSCs	PDX1	EGF, B27, GLP-1, betacellulin, HGF, nicotinamide, β-mercaptoethanol	Adoption of β-cells morphology and marker expression, insulin and C-peptide secretion
(Lima et al. 2013)	human exocrine pancreas-derived MSCs	PDX1-MAFA-NGN3-PAX4	betacellulin, exendin-4, nicotinamide	Insulin secretion
(Chun et al. 2015)	human amniotic fluid-derived MSCs	PDX1	activin A, β-mercaptoethanol, N2, B27, bFGF, nicotinamide	Expression of β-cells markers, insulin, and C-peptide secretion
(Xu et al. 2017)	human umbilical cord MSCs	PDX1-PAX4	B27	Acquisition of β-cells phenotype, expression of endocrine markers, insulin secretion
(Gao et al. 2018)	human adipose tissue-derived MSCs	PDX1	N/A	Insulin secretion

Early efforts directed towards obtaining β-cells, focused on embryonic stem cells (ESCs), taking advantage of the robust pluripotency of ECSs and established knowledge about natural β-cell genesis (Lumelsky et al., 2001). ESCs are guided towards definitive endoderm, then pancreatic progenitors, followed by endocrine progenitors, and finally mature β-cells. These protocols exploit culture conditions to guide the differentiation process into insulin-producing cells while concurrently using forced expression of the transcriptional factors regulating β-cell identity (Hebrok, 2012). D'Amour et al. (2005) was one of the first groups that differentiated ESCs into endoderm derivatives using Activin A in low serum content. Since then, various protocols have emerged with different modifications, changing signaling molecules, transcription factors, and culture conditions. Cho et al. (2008) have shown that exposure of human ESCs to betacellulin and nicotinamide alongside other factors sustains *PDX1* expression and further induces β-cell differentiation. This approach of stimulating *PDX1* expression indirectly was also adapted by Salguero-Aranda et al. (2016). Exposing mouse ESCs to diethylenetriamine nitric oxide adduct leads to enhanced expression of *Pdx1* when combined with valproic acid, and p300 inhibitor, enhanced pancreatic lineage specification and the generation of glucose-responsive insulin-producing cells (Salguero-Aranda et al., 2016). Another approach is the forced expression of *PDX1* by viral transduction, using adenoviral transduction of *PDX1*+*MAFA* and either *NGN3* or *NEUROD* alongside small molecules in a three-step protocol that guided the differentiation of mouse ESCs into insulin- and C-peptide—producing cells with elevated expression of β-cell markers (Xu et al., 2013). However, many of the obtained cells are not considered “true” β-cells mainly because they are immature, multihormonal, and unable to regulate glucose levels.

The emergence of induced pluripotent stem cells (iPSCs), circumvented ethical considerations related to ESCs and opened doors to generate patient-specific therapeutic cells. As with ESCs, differentiation protocols for iPSCs follow the same developmental stages as β-cells, beginning with definitive endoderm and moving towards pancreatic progenitors and differentiated β-cells. PDX1 plays a significant role in the differentiation of iPSCs into islet β cells, manifested by its activation of *NGN3* and *PAX6* expression after binding to their promoter regions and activating downstream gene networks (Qin et al., 2015). (Walczak et al., 2016) showed that lentiviral transduction of iPSCs with PDX1 and NKX6.1 induced the formation of insulin-producing cells with a morphology resembling β-cells, moreover, the derived cells secreted C-peptide in a glucose-responsive manner (Rajaei et al., 2018). Alongside PDX1, two other factors are usually used to push cells into β-cell differentiation. These are MAFA and NGN3. Together all three factors: PDX1, MAFA, and NGN3 are known as the PMN factors and play a critical role in β-cell differentiation and maturation. They have been used together in many research projects to obtain β-cells (Zhu Y. et al., 2017). For example, one study used a system to express the PMN factors in iPSCs under the control of a vanillic acid-dependent switch, coupled with a synthetic signaling cascade. Doing so allowed precise replication of the dynamics of endogenous expression and resulted in β-like cells comparable to human pancreatic islets in GSIS (Saxena et al., 2016). Differentiation protocols have witnessed many variations allowing the generation of cell populations called stem cell-derived islets (SC-islets) (Hogrebe et al., 2020; Maxwell and Millman, 2021). Although promising, generating human iPSCs remains inefficient and expensive, thus hindering the generation of large numbers of patient-specific lines.

Another promising source from which to obtain β-cells is mesenchymal stem cells (MSCs). MSCs have manifold advantages, like immunomodulation, which reduce immune system activation and anti-inflammatory factor release after transplantation (Scuteri and Monfrini, 2018). Transfection of bone marrow-derived MSCs with a recombinant plasmid harboring *PDX1* prompted islet-like structure formation, insulin secretion, and the adoption of β-cell morphology (Yuan et al., 2010). Furthermore, the level of *PDX1* expression is closely correlated with the level of insulin mRNA and the level of insulin secretion in differentiated, stable MSC cell lines (Yuan et al., 2012). Similar results have been shown in amniotic-fluid-derived MSCs upon adenoviral transduction with PDX1 in controlled culture conditions. Such MSCs were able to express β-cell markers, and secrete insulin and C-peptide (Chun et al., 2015). Similarly, umbilical-cord-derived-MSCs formed islet-like structures containing β-like cells that produced insulin, C-peptide, and other endocrine markers after delivery of *PDX1* (He et al., 2011) or the co-delivery of *PDX1* and *PAX4* using recombinant adenovirus under controlled media conditions (Xu et al., 2017).

Using small molecules and controlled culture conditions are of great importance in obtaining β-cells from other cell types. Lima et al. (2013) obtained functional β-like-cells from pancreatic-exocrine-tissue-derived MSCs after transduction with adenovirus harboring *PMN + PAX4*. Interestingly, the removal of serum from the media, stopping epithelial to mesenchymal transition (EMT), with Rho-associated kinase (ROCK), and the addition of small molecules like betacellulin, exendin-4 and nicotinamide resulted in better insulin expression and glucose regulation (Lima et al., 2013). Human adipose-tissue-derived-MSCs could also be differentiated into functional islet-like cells after transduction with adenovirus harboring *PDX1*. Differentiated cells secreted insulin and could regulate glucose in diabetic mice (Gao et al., 2018). All things considered, MSCs are great candidates for the treatment of diabetes, however, more studies are needed to evaluate the long-term differentiative capabilities of MSCs and their safety in application.

Soon after breaking the rigidity of adult differentiated cell types, new research focused on reprogramming mature cells directly into insulin-producing cells without inducing pluripotency. Obtaining β-cells from terminally differentiated cells might be a well-rounded alternative to the use of stem cells, considering their abundance and low risk of tumorigenesis, especially when sharing common progenitors with β-cells. Akinci et al. (2012) introduced the PMN factors using an adenoviral vector into 8 cell types, and demonstrated a bigger likelihood of developmentally related cells to transdifferentiate into β-cells. Of the 8 cell types studied, pancreatic exocrine and SOX9+ hepatic cells upregulated β-cell markers and secreted insulin (Akinci et al., 2012; Akinci et al., 2013).

Liver cells are developmentally related to β-cells and they could be reprogrammed to adopt β-cell morphology and markers. Much research has attempted to carry out *in vivo* differentiation in animal subjects starting from liver cells. For example, Ferber et al. (2000) identified the role of PDX1 in transforming murine liver cells into insulin-secreting cells after adenoviral transduction. The same results were repeated by Wang et al. (2007) who used plasmids harboring *PDX1* or *NGN3* with an unrelated adenovirus expressing the human coagulation factor *IX* gene (*AdVhFIX*). Diabetic mice treated with either of the two plasmids showed reduced hyperglycemia. Interestingly, when either of the two transcription factors was administrated in adeno-associated viral construct (AAV) there was no response in glucose levels. Later, when another group evaluated the effect of the hydrodynamic delivery of different plasmids carrying PMN factors in the livers of rats, they found that some plasmids like CpG-depleted plasmid (pCpG) and increased the levels of insulin mRNA up to 50 fold. Moreover, they detected signs of differentiation towards β-cells that were able to control glucose in hyperglycemic rats for 1 week (Cim et al., 2012). Similar results were obtained by Banga et al. (2012), who delivered PMN factors into murine livers by adenoviral transduction and obtained glucose-sensitive cells that ameliorated diabetes in mice. Hepatic to pancreatic differentiation could also be achieved by ectopic expression of *PDX1* by recombinant adenovirus (Ber et al., 2003).

Some studies have used the active form of PDX1 (PDX1-VP16) in which the activation domain from herpes simplex virus (VP16) is fused to the C-terminus of PDX1, which allows PDX1 to activate target genes without association with other co-factors (Horb et al., 2003). Compared to PDX1, PDX1-VP16 is more efficient in initiating liver-to-endocrine pancreas differentiation, however, both of them upregulated β-cell genes and reversed hyperglycemia in diabetic mice upon lentiviral transduction (Tang et al., 2006). Imai et al. (2005) showed that adenoviral transduction with PDX1-VP16 induced murine hepatocytes to produce insulin and to regulate hyperglycemia in diabetic mice, however, the expression pattern of the hepatocyte was maintained. Kaneto et al. (2005) also showed that PDX1-VP16 increased insulin and other pancreatic factors in the livers of mice and controlled hyperglycemia, especially when combined with NEUROD or NGN3. Cao et al. (2004) showed that the complete transdifferentiation of hepatic cells into insulin-producing cells by PDX1-VP16 requires additional external factors like high glucose and hyperglycemia. Introducing exogenous PDX1-VP16 protein into definitive endoderm cells generated from ESCs, mimicking the natural pattern of *PDX1* expression, induced an endocrine pancreas-like cell phenotype, in which 30% of the cells were β-like cells (Bernardo et al., 2009). Taking into account that the PDX1-VP16 approach showed convincing results in the conversion of ESCs and hepatic cells into β-like cells, perhaps this approach can be also adapted for the transdifferentiation of other adult cells into β-cells.

*In vivo* studies have also been performed on other cell types. Zhou et al. (2008) used adenovirus to deliver PMN into differentiated pancreatic exocrine cells. The reprogrammed cells resembled β-cells morphologically and functionally. Another study screened the effect of PMN factors in a variety of tissues. They found that expression of PMN in the intestinal crypts gives them β-like features including glucose sensitivity and insulin secretion ability (Chen et al., 2014).

α-cells have been particularly well-studied as sources for β-cell compensation due to their sharing of a close progenitor. The differential plasticity between alpha and β-cells was proven to be subject to the effects of PDX1. Introducing PDX1 in NGN3+ endocrine progenitors in the embryonic period in mice resulted in rapid postnatal reprogramming of α-cells to insulin-positive cells resembling β-cells (Yang et al., 2011). Transduction of AAV carrying *PDX1* and *MAFA* into the pancreas of diabetic mice leads to increased α-to β-cell transdifferentiation, increased glucose responsiveness, and normalized blood sugar (Xiao et al., 2018). Moreover, repression of α-cell genes like *ARX* while upregulating *PAX4* is another approach to guide α-to β-cells differentiation. Lima et al. (2016) used PMN factors alongside PAX4 and siRNA against ARX in the exocrine pancreas to favor the formation of β-over α-cells. The obtained β-like cells efficiently processed and secreted insulin and were able to respond to glucose and normalize its levels in diabetic mice.

Gall bladder epithelial cells have also been studied in this regard, after adenoviral transduction with PMN factors. The reprogrammed cells upregulated β-cell genes while downregulating epithelial genes, however, they were not true functional β-cells because they were non-responsive to glucose despite their insulin secretion ability (Hickey et al., 2013). Cardinale et al. (2015) identified a heterogeneous stem/progenitor cell population in the human biliary tree that, when exposed to PDX1 protein in the media, internalized the protein and started insulin and C-peptide production, indicating endocrine differentiation. Another group upregulated the expression of *PDX1* indirectly by identifying an andrographolide named C1037 that can stimulate *PDX1* expression in both its resulting mRNA and protein levels. Pancreatic duct cells treated with C1307 increased expression of insulin while decreasing glucagon levels compared to control groups (Zhang et al., 2020).

The numerous variations of differentiation protocols rule out the existence of a unified method and source for obtaining β-cells. Although promising, this numerosity indicates the need for more studies and clinical trials to find a unified protocol involving the least amount of genetic and cellular manipulation. All the protocols summarized in this review have focused on the activation of important β-cell genes, primarily *PDX1*. The methods of activation alongside culture conditions and added small molecules can vary and affect the differentiation outcome. Some protocols rely solely on small molecules to provide conditions similar to β-cell formation *in vivo*, which avoids problems associated with viral transduction. Choosing the right cell of origin can mean the difference between a complex or a simple protocol. As mentioned before, cells that share a common progenitor with β-cells are easier to transform. Stem cells have great pluripotency potential but this raises the risk of tumorigenesis. In conclusion, this data shows the importance of PDX1 in obtaining β-cells considering its role as a master regulator of β-cell function and identity.

## 7 Conclusion

Numerous studies on PDX1 have demonstrated its critical role in organogenesis, differentiation, maturation, and in maintaining, and preserving β-cell identity. Moreover, much research has accomplished the differentiation of non-β-cells into insulin-producing cells with the aid of PDX1 as a master regulator in the differentiation protocols, or alongside other factors and soluble molecules. This property of PDX1 makes it a particularly important target for gene or replacement therapy approaches to the treatment of diabetes. Our increased knowledge of PDX1 and other pancreatic endocrine factors provides the cornerstone for optimizing differentiation protocols. Through the years, these protocols have witnessed many advances and variations, including various combinations of transcription factors, culture media components, and different source cells. However, we are still far from finding a bona fide alternative to human β-cells, but our understanding of the role of transcription factors, their interactions and intricate regulation by each other together with the further impact of small molecules and epigenetic factors should eventually allow us to obtain functional β-cells that would be suitable for transplantation therapy in the treatment of diabetes.

## Nomenclature

### Abbreviations

AAV: Adeno-associated virus
bFGF: basic fibroblast growth factor
EMT: Epithelial–mesenchymal transition
ESC: embryonic stem cells
GPCR: G protein-coupled receptor
HSS: Nuclease hypersensitive site
iPSCs: induced pluripotent stem cells
ITS: insulin–transferrin–selenium
lncRNA: long non-coding RNA
MODY: Maturity-onset diabetes of the young
MSC: mesenchymal stem cells
ncRNA: non-coding RNA
PMN: PDX1, MAFA, NGN3
RA: retinoic acid
ROCK: Rho-associated kinase
SC-islets: stem cells–derived islets
Shh: Sonic hedgehog
siRNA: Small interfering RNA
SIRT: sirtuin
T1D: Type 1 diabetes
T2D: Type 2 diabetes
TF: Transcription factor

### Genes and proteins

AdVhFIX: Human coagulation factor IX gene
AKT: RAC-alpha serine/threonine-protein kinase
ARX: Aristaless related homeobox
CDX2: Caudal Type Homeobox 2
CK2: Casein kinase II
FAM3A: FAM3 metabolism regulating signaling molecule A
FGF: Fibroblast growth factor
FGF2: Fibroblast growth factor 2
FOXA1: Forkhead box A1
FOXA2: Forkhead box A2
GAS5: growth arrest-specific transcript 5
GATA4: GATA Binding Protein 4
GCK: Glucokinase
GLUT2: Glucose transporter 2
GRG3: Groucho-related gene 3
GSIS: Glucose-stimulated insulin secretion
HDAC1: Histone deacetylase 1
HDAC2: Histone deacetylases 2
HES1: hes family bHLH transcription factor 1
HNF1α: Hepatocyte nuclear factor 1 α
HNF3β: Hepatocyte nuclear factor 3 β
HNF4β: Hepatocyte nuclear factor 4 β
HNF6: Hepatocyte nuclear factor 6
IAPP: Islet amyloid polypeptide
IGF1: Insulin-like growth factor 1
INS1: Insulin I
IPF1: Insulin-promoting factor 1
MAFA: MAF bZIP transcription factor A
MAFB: MAF bZIP transcription factor B
NEUROD: Neuronal differentiation 1
NGN3: Neurogenin-3
NKX2.2: NK2 Homeobox 2
NKX6.1: NK6 Homeobox 1
OC1: One Cut Homeobox 1
PAX4: Paired box 4
PAX6: Paired box 6
PDX1: Pancreatic and duodenal homeobox 1
PLUTO: PDX1 locus upstream transcript
PTF1α: Pancreas-specific transcription factor 1α
RFX6: Regulatory factor X6
SLC2A: Solute carrier family 2 member 9
SOX17: SRY-Box transcription factor 17
SOX9: SRY-Box transcription factor 9
SST: Somatostatin
UCN3: Urocortin 3
USF1: Upstream stimulatory factor 1
