# Supplementary material for: PDX1 is the cornerstone of pancreatic β-cell functions and identity
Source: Front Mol Biosci. 2022 Dec 15;9:1091757. doi: 10.3389/fmolb.2022.1091757 (PMC9798421; doi:10.3389/fmolb.2022.1091757)
Supplement: Supplementary file 1 [file Table1.DOCX]

| **Table 1: summary of studies using PDX1 in obtaining β-cells** | | | | | |
| --- | --- | --- | --- | --- | --- |
| **study** | **cell source** | **transcription factors** | **small molecules** | **medium** | **outcome** |
| **differentiated adult cells** | | | | | |
| (Ferber et al., 2000) | mouse liver cells | Ad**- PDX1** | N/A | *in vivo*, (into the liver of BALB/C and C57BL/6 mice) | increased hepatic and plasma immunoreactive insulin content, amelioration of hyperglycemia in diabetic mice. |
| (Ber et al., 2003) | mouse liver cells | Ad**- PDX1** | N/A | *in vivo*, (into the liver of BALB/C and mice) | induction of both pancreatic exocrine and endocrine genes, including insulin which ameliorated diabetes in mice |
| (Imai et al., 2005), (Kaneto et al., 2005),(Cao et al., 2004), (Tang et al., 2006) | mouse liver cells | **PDX1**-VP16  **PDX1**  (different means of transduction, transfection) | N/A | *in vivo*, (into the liver of mice) | PDX1-VP16 induced insulin expression and other pancreatic markers, reversed hyperglycemia in mice |
| (Wang et al., 2007) | mouse liver cells | **pPDX1**+ AdVhFIX  or  pNGN3+ AdVhFIX | N/A | *in vivo,* (into the liver of C57/Bl6 mice) | amelioration of diabetic mice, near-normal GSIT function |
| (Zhou et al., 2008) | mouse pancreatic exocrine cells | Ad-(**PDX1**- MAFA-NGN3) | N/A | *in vivo*, (into the pancreas of adult mice) | differentiated cells resemble β-cells morphologically, express β -cells markers, and can ameliorate hyperglycemia by secreting insulin |
| (Banga et al., 2012) | mouse hepatic duct-like cells | Ad- (**PDX1**-MAFA- NGN3) | N/A | *in vivo,* (liver of NOD-SCID mice) | reprogrammed cells adopted β-cell morphology and marker expression; they were also able to relieve diabetes in mice |
| (Cim et al., 2012) | rat liver cells | pCpG-**PDX1**, pCpG-NGN, pCpG-MAFA | N/A | *in vivo,* (hydrodynamic delivery in the liver of rats) | increased expression of insulin mRNA |
| (Hickey et al., 2013) | mouse gall bladder epithelial cells | Ad-(**PDX1**-MAFA-NGN3) | 1 day after transduction: 2 µM retinoic acid, 1% DMSO  2 days after transduction:  250 nM of γ-secretase inhibitor dibenzazepine (DBZ), 1% DMSO | DMEM,  15% FBS, antimicrobials | upregulation of β-cells genes and downregulation of gall bladder epithelial genes, low glucose responsivity, survival *in vivo* and insulin secretion |
| (Akinci et al., 2013) | rat pancreatic exocrine cells and hepatocytes,  mouse hepatocyte-derived small cells | Ad-(**PDX1**-MAFA-NGN3) | 2 days before viral transduction: DAPT 10 μM, G9a HMT enzyme inhibitor BIX-01294 2 μM,  adenosine agonist NECA 10 μM | DMEM, 10% FBS, 2mM L-glutamine, antimicrobials | increased expression of β-cell markers and insulin secretion, small molecules increased the number of insulin-positive cells by 6 folds |
| (Chen et al., 2014) | mouse intestine crypts cells | **PDX1**-MAFA-NGN3 | N/A | *In vivo,* (double transgenic mice) | expression of transgenes in the intestinal crypts gives them β-like-cells features, amelioration of diabetes in mice |
| (Cardinale et al., 2015) | human biliary tree stem cells | recombinant **PDX1** in the form of a fusion protein was added to the medium at 0.1 μM concentration | Modified Kubota’s Medium (MKM),  Serum-free, calcium (0.6 mM), copper (10–12 M) and 20 ng/ml bFGF, with PDX-1 peptide 0.1 μM | | increased expression of β-cells markers, insulin and C-peptide secretion |
| (Lima et al., 2016) | human exocrine pancreatic cells | recombinant Ad-mouse (**PDX1**-NGN3-MAFA- PAX4) / at day 4  Transfection with 4 siRNA against ARX using transfection reagent Dharmafect 1/ at day 6 | RPMI 1640, serum-free,  1%BSA, low glucose, culture on laminin-coated plates.  first stage for 3 days:  insulin-transferrin-selenium,  1 μM 5-aza-2'-deoxycytidine,  1 mM sodium butyrate, 10 μM SB431542, 2 μM Y27632  second stage for 6 days:  1 nM betacellulin, 10 nM exendin-4, 10 mM nicotinamide | | obtained cells efficiently processed, packaged, and secreted insulin, normalization of glucose levels in diabetic mice |
| (Xiao et al., 2018) | mouse pancreatic alpha cells | AAV-serotype 8 (**PDX1**- MAFA) | N/A | *in vivo* | increased β-cell mass, prolonged normalized blood glucose |
| **embryonic stem cells** | | | | | |
| (Xu et al., 2013) | mouse ESCs | pAd **PDX1**-nGFP + pAd MafA-I-nGFP + pAd NeuroD-I-nGFP or pAd Ngn3-I-nGFP/ at third or second step | First step:  2 days in D-MEM,15%FCS, 2 mM l-glutamine, nonessential amino acids, antimicrobials,100 μM β-mercaptoethanol.  2 days in serum-free X-VIVO 10 medium, 100 ng/ml activin A  1 day in DMEM, 2% FBS, 2 mM retinoid acid, 1% ITS  Second step:  5 days in: DMEM low glucose, 10% FBS, 10 ng/ml bFGF, 20 ng/ml EGF, 1% ITS  Third step:  5 days in DMEM/F12, 1% N2, 1% B27, 10 ng/ml bFGF, 1% ITS,10 mM nicotinamide | | increased expression of β-cells markers after the final stage, transcription factors dramatically increased expression of insulin gene and insulin secretion in response to glucose |
| (Salguero-Aranda et al., 2016) | mouse ESCs | increased ***PDX1*** expression by small molecules | 19 h: 500 μM DETA-NO  6 days: 100 μM valproic acid  20 h: 50 μM P300 inhibitor C646 | DMEM, 15% FBS, 0.1 mM β-mercaptoethanol, 2 mM l-glutamine, 1% minimum essential medium (MEM) nonessential amino acids, antimicrobials | increased expression of β-cells markers, glucose responsivity, and insulin secretion |
| **induced pluripotent stem cells** | | | | | |
| (Saxena et al., 2016) | human iPSCs | Activation of **PDX1**, MAFA, and NGN3 under the vanillic acid-controlled switch | Step 1: 24h, RPMI, Activin A, Wnt3A 48h, RPMI, Activin A, bFGF, BMP-4, VEGF 48h, serum-free differentiation (SFD) medium, 1-thioglycerol, Activin A, bFGF, BMP-4, VEGF165  Step 2, 72:h SFD medium, Wnt3A, noggin, FGF-10  Step 3, 72 h: DMEM, 1% vitamin A-free B−27 serum-free supplement, ascorbic acid, KAAD-cyclopamine, retinoic acid, FGF-10  Step 4, 72 h: DMEM, 1%vitamin A-free B−27 serum-free supplement, ascorbic acid, retinoic acid, FGF-7, EGF  Step 5, 4 h: Y-27632 72 h: in suspension, plates to facilitate the formation of aggregates in DMEM containing 1% vitamin A-free B−27 serum-free supplement, ascorbic acid, retinoic acid, thyroid hormone T3, Alk5 inhibitor, noggin, KAAD-cyclopamine, gamma-secretase inhibitor  Step 6, 72 h: DMEM containing 1% vitamin A-free B−27 serum-free supplement, ascorbic acid (50 μg ml−1), retinoic acid, thyroid hormone T3, noggin, Alk5 inhibitor, and gamma-secretase inhibitor  Step 7, 5 days: low-glucose DMEM, 10% FCS, 1 × non-essential amino acid solution, thyroid hormone T3, Alk5 inhibitor | | Differentiated cells resemble mature β-cells and secreted insulin upon glucose stimulation |
| (Rajaei et al., 2018) | human iPSCs | LV-**PDX1** | First stage 1 day:  RPMI, serum-free, activin A (100 ng/ml), Wnt3a (25 ng/ml)  Second stage 2 days:  RPMI, 0.2% FBS, activin A (100ng/ml).  Third stage 3 days: RPMI, 2% FBS, KGF (50 ng/ml), SB431542 (2.5 μM)  Fourth stage 3 days: DMEM, 1% B27, KAAD cyclopamine (0.25 μM), RA, 2 μM, noggin (50ng/ml) for another 3 days.  Fifth stage 3 days: DMEM, 1% B27, Noggin (50ng/ml), KGF (50ng/ml), EGF (50ng/ml) for the next 3 days.  Sixth stage 3 days: DMEM, 1% B27, Noggin (50 ng/ml), KGF (50 ng/ml), EGF (50 ng/ml), and IBMX (100 μM) | | Generation of insulin-producing glucose-responsive cells with β-cell morphology and C-peptide secretion |
| **mesenchymal stem cells** | | | | | |
| (Yuan et al., 2010) | rat bone marrow-derived MSCs | pEGFP- **PDX1**  pcDNA3.1-**PDX1**  transfection by lipofectamine | First step (pre-induction) 3 days:  DMEM, serum-free, Low glucose 5.5 mM, 1% DMSO  Second step (induction) 9 days:  DMEM, 10% FBS, high glucose 25 mM, 1% DMSO | | MSCs formed islet-like structures resembling β-cells phenotype and secreted insulin |
| (He et al., 2011) | human umbilical cord MSCs | rAd-**PDX1** | Stage 1, 7 days: viral transduction, DMEM/F12, 10% FBS Stage 2, 3 days: DMEM/F12, 2% FBS, 100ng/ml EGF, 2% B27 Stage 3, 7 days: addition of 10 ng/ml GLP-1, 10 ng/ml betacellulin, 10 ng/ml HGF, 10 mmol/l nicotinamide, 2% B27, 0.1 mmol/l β-mercaptoethanol | | MSCs adopted β-cell morphology and phenotype, increased expression of both insulin and C-peptide |
| (Lima et al., 2013) | human exocrine pancreas-derived MSCs | Ad- **PDX1**, MaFA, NGN3, PAX4 | RPMI 1640, serum free, of 1 nmol/L betacellulin, 10 nmol/L exendin-4, and 10 mmol/L nicotinamide  chromatin-modifying agents,  small molecules that inhibit EMT | | insulin secretion in response to glucose, normalizing glucose in diabetic mice |
| (Chun et al., 2015) | human amniotic fluid-derived MSCs | Ad-**PDX1** serotype 5 | First step 48 h:  adenovirus transduction,  a-MEM,15% FBS, 1% glutamine and 1% antibiotics, 18% Chang B, 2% Chang C  second step 24-48 h:  replating on matrigel in the same medium  third step 24-48 h:  addition of activin A (100 ng/ml)  fourth step: transfer to coated plates in knockout-DMEM with 0.1 mM β-mercaptoethanol, 1% antibiotics, 1% L-glutamine, 1% N2, 2% B27, 1% non-essential amino acids and 25 ng/ml bFGF. After 24–48 h, 10 mM nicotinamide was added. | | enhanced expression of β-cells markers, insulin and C-peptide secretion |
| (Xu et al., 2017) | human umbilical cord MSCs | rAd **PDX1**  rAd **PDX1**+PAX4 | B27(concentration not mentioned) | DMEM/F12  FBS (concentration not mentioned) | Acquisition of islet-like structures, expression of insulin and other endocrine markers |
| (Gao et al., 2018) | human adipose tissue-derived MSCs | Ad-**PDX1** | Not mentioned | | insulin secretion, normalizing glucose in diabetic mice |

**Abbreviations**

AAV - Adeno-associated virus

bFGF - basic fibroblast growth factor

EMT -  Epithelial–mesenchymal transition

ESC - embryonic stem cells

GPCR - G protein-coupled receptor

HSS - Nuclease hypersensitive site

iPSCs - induced pluripotent stem cells

ITS - insulin–transferrin–selenium

lncRNA - long non-coding RNA

MODY - Maturity-onset diabetes of the young

MSC - mesenchymal stem cells

ncRNA - non-coding RNA

PMN - PDX1, MAFA, NGN3

RA - retinoic acid

ROCK - Rho-associated kinase

SC-islets - stem cells – derived islets

Shh - Sonic hedgehog

siRNA - Small interfering RNA

SIRT – sirtuin

T1D - Type 1 diabetes

T2D - Type 2 diabetes

TF - Transcription factor

**Genes and proteins:**

AdVhFIX - Human coagulation factor IX gene

AKT - RAC-alpha serine/threonine-protein kinase

ARX - Aristaless related homeobox

CDX2 - Caudal Type Homeobox 2

CK2 - Casein kinase II

FAM3A - FAM3 metabolism regulating signaling molecule A

FGF - Fibroblast growth factor

FGF2 - Fibroblast growth factor 2

FOXA1 - Forkhead box A1

FOXA2 - Forkhead box A2

GAS5 - growth arrest-specific transcript 5

GATA4 - GATA Binding Protein 4

GCK - Glucokinase

GLUT2 - Glucose transporter 2

GRG3 - Groucho-related gene 3

GSIS - Glucose-stimulated insulin secretion

HDAC1 - Histone deacetylase 1

HDAC2 - Histone deacetylases 2

HES1 - hes family bHLH transcription factor 1

HNF1α - Hepatocyte nuclear factor 1 α

HNF3β - Hepatocyte nuclear factor 3 β

HNF4β - Hepatocyte nuclear factor 4 β

HNF6 - Hepatocyte nuclear factor 6

IAPP - Islet amyloid polypeptide

IGF1 - Insulin-like growth factor 1

INS1 - Insulin I

IPF1 - Insulin-promoting factor 1

MAFA - MAF bZIP transcription factor A

MAFB - MAF bZIP transcription factor B

NEUROD - Neuronal differentiation 1

NGN3 - Neurogenin-3

NKX2.2 - NK2 Homeobox 2

NKX6.1 - NK6 Homeobox 1

OC1 - One Cut Homeobox 1

PAX4 - Paired box 4

PAX6 - Paired box 6

PDX1 - Pancreatic and duodenal homeobox 1

PLUTO - PDX1 locus upstream transcript

PTF1α - Pancreas-specific transcription factor 1α

RFX6 - Regulatory factor X6

SLC2A - Solute carrier family 2 member 9

SOX17 - SRY-Box transcription factor 17

SOX9 - SRY-Box transcription factor 9

SST - Somatostatin

UCN3 - Urocortin 3

USF1 - Upstream stimulatory factor 1
